# Nano-Scale Morphology of Melanosomes Revealed by Small-Angle X-Ray Scattering

**DOI:** 10.1371/journal.pone.0090884

**Published:** 2014-03-12

**Authors:** Thomas Gorniak, Tamas Haraszti, Vasyl M. Garamus, Andreas R. Buck, Tobias Senkbeil, Marius Priebe, Adam Hedberg-Buenz, Demelza Koehn, Tim Salditt, Michael Grunze, Michael G. Anderson, Axel Rosenhahn

**Affiliations:** 1 Institute of Functional Interfaces (IFG), Karlsruhe Institute of Technology (KIT), Eggenstein-Leopoldshafen, Germany; 2 Applied Physical Chemistry, Ruprecht-Karls-University Heidelberg, Heidelberg, Germany; 3 Analytical Chemistry - Biointerfaces, Ruhr-University Bochum, Bochum, Germany; 4 Max-Planck-Institute for Intelligent Systems, Stuttgart, Germany; 5 Helmholtz-Zentrum Geesthacht, Zentrum für Material- und Küstenforschung GmbH, Geesthacht, Germany; 6 Institute for X-Ray Physics, University of Göttingen, Göttingen, Germany; 7 Department of Molecular Physiology and Biophysics, The University of Iowa, Iowa City, Iowa, United States of America; 8 Center for the Prevention and Treatment of Visual Loss, Iowa City Veterans Affairs (VA) Health Care System, Iowa City, Iowa, United States of America; USGS National Wildlife Health Center, United States of America

## Abstract

Melanosomes are highly specialized organelles that produce and store the pigment melanin, thereby fulfilling essential functions within their host organism. Besides having obvious cosmetic consequences – determining the color of skin, hair and the iris – they contribute to photochemical protection from ultraviolet radiation, as well as to vision (by defining how much light enters the eye). Though melanosomes can be beneficial for health, abnormalities in their structure can lead to adverse effects. Knowledge of their ultrastructure will be crucial to gaining insight into the mechanisms that ultimately lead to melanosome-related diseases. However, due to their small size and electron-dense content, physiologically intact melanosomes are recalcitrant to study by common imaging techniques such as light and transmission electron microscopy. In contrast, X-ray-based methodologies offer both high spatial resolution and powerful penetrating capabilities, and thus are well suited to study the ultrastructure of electron-dense organelles in their natural, hydrated form. Here, we report on the application of small-angle X-ray scattering – a method effective in determining the three-dimensional structures of biomolecules – to whole, hydrated murine melanosomes. The use of complementary information from the scattering signal of a large ensemble of suspended organelles and from single, vitrified specimens revealed a melanosomal sub-structure whose surface and bulk properties differ in two commonly used inbred strains of laboratory mice. Whereas melanosomes in C57BL/6J mice have a well-defined surface and are densely packed with 40-nm units, their counterparts in DBA/2J mice feature a rough surface, are more granular and consist of 60-nm building blocks. The fact that these strains have different coat colors and distinct susceptibilities to pigment-related eye disease suggest that these differences in size and packing are of biological significance.

## Introduction

Melanosomes [1] are organelles that contain melanin [2] and fulfill important biochemical tasks in their host cells. They are responsible for the color of hair, skin and the iris, provide protection against the damaging effects of UV radiation and scavenge free radicals [3]. Variations in melanosome structure and function are also associated with multiple diseases, including several forms of albinism [4], cancer [5], and eye disease [6]. Melanosomes have been the subject of numerous microscopy studies aiming to provide insights into their roles in health and disease [7], [8] However, the exceptional electron density of melanin has been an obstacle in probing the ultrastructure of melanosomes by traditional approaches such as transmission electron microscopy (TEM). Thus, melanosomes are increasingly being studied using specialized approaches such as 3D electron tomography [9] and STXM (scanning transmission X-ray microscopy) [10], [11]. Also, methods like XANES (X-ray absorption near-edge spectroscopy) [12] and AFM (atomic-force microscopy) [13]–[15] have revealed chemical states and surface structures, respectively. Data from each of these approaches have led to new insights into aspects of melanosome ultrastructure that had previously been impervious to enquiry.

Small-angle X-ray scattering (SAXS) is a technique whereby a sample is exposed to a focused X-ray beam and the scattered radiation is registered by a detector [16]. Analyzing X-ray scattering can yield structural information – particularly with respect to average size, shape, ultrastructure and domain orientation – on a nanometer scale [17]–[20]. It allows biological materials to be studied in a largely natural state, and with relatively minimal sample preparation. Here, we utilize SAXS to study iridial melanosomes from inbred strains of mice by a multiscale approach [21], [22], and report genotype-specific differences in their granularity and surface morphology. Though the pigment melanin in its isolated form has already been studied by SAXS [23]–[26], to our knowledge the experiments presented here are the first to provide structures of whole, physiologically intact melanosomes based on this technique.

## Materials and Methods

### 2.1. Ethics statement

This study was carried out in strict accordance with the recommendations in the Guide for the Care and Use of Laboratory Animals of the National Institutes of Health. The protocol was approved by the Institutional Animal Care and Use Committee of the University of Iowa (Animal Protocol 1006131). All mice were euthanized in accordance to the “AVMA guidelines on Euthanasia”.

### 2.2. Sample preparation

Melanosomes were extracted from 6-week old C57BL/6J (B6) and DBA/2J (D2) mice, according to the protocol described in [10]. In brief, immediately after animals were euthanized by cervical dislocation, the eyes were enucleated and placed into ice-cold buffer (pH 7.2, 10 mM HEPES and 0.25 M sucrose). The anterior chambers were then dissected by circumferential incision just posterior of the limbus. Using forceps, the lens was removed, granting access to the iris. Irides were detached from the ciliary body and placed into fresh buffer and homogenized using an ice-cold 7-ml Dounce glass∶glass homogenizer (Kimble–Kontes), 50 strokes of the A pestle and 30 strokes of the B pestle were applied.

The homogenized samples were centrifuged at 800 g for 10 min, at a temperature of 5°C. The supernatant, which contained the melanosomes, was removed and centrifuged at 15 000 g for 15 min, while the temperature was held at 5°C. The pelleted organelles were resuspended in fresh buffer for storage and later used in experiments. The organelles extracted from each mouse strain were pooled separately. Hence, each sample used for the experiments consisted of a mixture of melanosomes from eight animals belonging to a group of the same strain and the same age.

In the case of SAXS measurements carried out at ambient temperatures, the organelles did not require treatment beyond that described above. However, the SAXS experiments at cryogenic temperatures required vitrification of the organelles on TEM grids (copper with a 200 mesh covered by a holey carbon C-flat film with a 2/4 perforation, Protochips Inc., USA). Samples were vitrified by pipetting 4 µl of organelle suspension onto a TEM grid, blotting the liquid with cellulose paper (Whatman grade No. 1) and using a plunge-freezer to quickly dip [27] the sample into a liquid mixture of 37% ethane and 63% propane, which produces a temperature of about 80 K [28]. The cryo-fixed samples were then stored in liquid nitrogen.

For the SEM experiments, the samples were freeze-dried using an apparatus built in house. The pressure during sublimation was set to 1×10^−8^ mbar. The specimen was allowed to warm from 80 K to 300 K over 48 h without regulation. Prior to freeze-drying, the organelles were prepared basically according to the vitrification process described above, but with two exceptions: special grids were used to support the samples (HZB-2, Gilder Grids, UK), and the melanosomes were washed with Milli-Q water before being subjected to cryo-fixation. The latter step was added because we found that during dehydration the components present in the buffer solution built up a tension force that disrupted the fragile carbon nano sheets, rendering the samples useless. The washing protocol was as follows: 10 µl of physiological organelle suspension was gradually diluted by the sequential addition of 30 µl, 120 µl and 800 µl Milli-Q water, with a 15 min relaxation period between each addition. The diluted suspension was then centrifuged at 14 000 g for 15 min at a temperature of 5°C, to pellet the melanosomes. The supernatant was removed and the melanosomes were resuspended in pure Milli-Q water.

In order to minimize the possible impact of osmotic stress, the organelle preparations were cryo-fixed immediately after the final resuspension. After dehydration was complete, an approximately 5-nm layer of carbon was applied using a vacuum coater (EM ACE200, Leica, Germany).

### 2.3. Small angle X-ray scattering at ambient temperatures

These experiments were carried out at the P12 BioSAXS beamline of the synchrotron radiation source PETRA III (EMBL/DESY) in Hamburg. Photons with an energy of *E* = 12.8 keV were selected using a double crystal monochromator, and focused to a spot of 0.2×0.1 mm^2^ (horizontal×vertical). The photon flux of the focused beam was 5×10^12^ ph/s. The sample suspension was delivered by an automatic sample robot, which drew liquid from the solution reservoir, into an actively cooled (10°C) glass capillary. A sample of approximately 20 µl was illuminated; this volume contained roughly 10^3^ melanosomes. The diffracted signal was recorded using a single-photon-counting PILATUS 2M detector (Dectris, Switzerland).

For each strain, we recorded 20 diffraction patterns originating from the same sample volume, using an exposure time of 0.05 s. Before and after each SAXS measurement from the melanosome suspension, signal from pure buffer was measured, and used for background subtraction. This background-corrected SAXS data were used to calculate one-dimensional scattering curves by angular averaging. In order to verify that no artifacts had occurred as a result of radiation damage, all scattering curves for a recorded dataset were compared to a reference curve (typically the first exposure) before being integrated using an automated acquisition and analysis program [29]. The range of the reciprocal space vector *q* was calibrated using diffraction patterns of silver behenate [30].

Data were analyzed by fitting to a theoretical model that had been developed to describe the scattering from surface and volume fractals [22], [31], and has been successfully applied to data from a variety of aggregate systems covering a wide range of length scales [32]–[35]. These systems share a common feature: plotting their scattering intensity *I* versus *q* in a double logarithmic plot yields curves with multiple straight sections. This theory's main advantage is its ability to model multiple adjacent straight sections simultaneously. This is accomplished by applying a linear combination of power law and exponential Guinier terms (as generalized by Beaucage), that represent surface and bulk scattering, respectively [21]. This approach avoids uncertainties that occur when each isolated straight section is separately fitted with the simple power law *I*(*q*) = *q* ^−*p*^.

The parameter *p* – which in a double logarithmic plot is also referred to as the slope of *I*(*q*) – contains information about the scattering object's surface structure if 3<*p*<4 (values close to 3 indicating a rough surface and those close to 4 a smooth surface), and about its volumetric properties if 1<*p*<3 (values starting from 1 and going to 3 correspond to a transition from a chain-like to a space-filling aggregate structure) [34]. These conclusions are valid for real space scale lengths *d* = 2*π*/*q* that correspond to scattering vectors *q* from an interval in which the power law can be applied. Thus, deviation from a straight line, i.e. a deflection or kink in the SAXS curve towards a smaller *q*-value, marks the length scale at which a component of the irradiated sample of a distinct size begins to dominate the scattering signal. The *q*-value at which the deflection occurs can be used to estimate the size of the corresponding object or subunit [35].

For the data from B6 mice, we confined our analysis to the *q*-interval from 0.07 nm^−1^ to 0.27 nm^−1^. Cutting-off the first four data points in the low *q*-regime removes possible artifacts resulting from proximity of the beamstop. The upper interval limit was chosen because it marks another kink. Its modeling would extend the number of independent parameters beyond a reasonable degree, rendering a meaningful fitting process impossible. Furthermore real-space length scales that correspond to *q*-values>0.27 nm^−1^ are *d*<20 nm, which is in the range of macromolecular entities. In the case of a complex organelle, we expect structural information on this scale to be highly ambiguous and unreliable. Hence, the model for the *q*-range of interest assumes two straight sections within the scattering amplitude, and is [22]

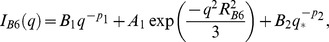
where *A*_1_, *B*_1_ and *B*_2_ are scaling parameters, *p*_1_ and *p*_2_ reflect the slopes of the straight sections and *R*_B6_ denotes the radius of gyration of the particles present. In brief, the signal within the inspected *q*-interval represents surface scattering from particles of *d*>125 nm (first term), volume scattering from particles of *d*<125 nm (second term) and surface scattering from the latter (third term). The asterisk in Eq. (1) indicates a rescaling of the third term according to
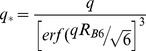



This incorporation of the Gaussian error function erf(·) ensures that the power law does not contribute to the signal at *q*-values that correspond to real-space scales >*R*_B6_.

The ansatz described above is termed the *model of independent scatterers*, which can be applied to data where the slope value *p* of adjacent straight sections in the measured signal *I*(*q*) becomes smaller when going from lower to higher *q*-values [34]. In the opposite case, where the slope value *p* increases, a more complex approach called the *model of dependent scatterers* must be applied.

For the organelles from the D2 mice, scattering intensities were better described by the latter approach for dependent scatterers. In this instance, both contributions (surface and volume scattering) in the low *q*-regime needed to be considered simultaneously. This is reflected by the product of a Guinier term, and a power law in the first addend of the function [22], [34]

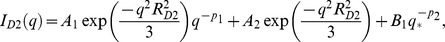
which takes into account the scaling factors *A*_1_, *A*_2_ and *B*_1_, the slopes *p*_1_ and *p*_2_ and the radius of gyration *R*_D2_.

### 2.4. X-ray scattering at cryogenic temperatures

These experiments were carried out at the GINIX nanofocus endstation of the P10 beamline [36] at the synchrotron radiation source PETRA III. Photons with an energy of *E* = 7.9 keV were focused, using a Kirkpatrick-Baez mirror pair [37], to a spot measuring 400 nm and 200 nm (full width at half maximum) in the horizontal and vertical directions, respectively. During the measurements, the sample was cooled using a 100 K nitrogen stream (Cryojet, Oxford Instruments, UK). We employed a single-photon-counting detector (PILATUS 300k, Dectris, Switzerland), which was placed 5.13 m downstream of the sample, to record two-dimensional diffraction patterns. The patterns were generated by scanning the sample through the focal spot at a rectangular step size of 400 nm. With an exposure time of 0.1 s per scanning point, the applied dose was approximately 3.4×10^6^ Gy per raster position. The PILATUS detector was protected from the direct beam by placing two semi-opaque beamstops between it and the sample.

From the recorded Fourier space signal at different sample positions, real-space images were calculated. The magnitude at each scan position was derived from the total number of photons scattered off the optical axis (*q*>0.05 nm^−1^), yielding images similar to darkfield STXM. Such a scanning experiment allows sampling the specimen with a resolution determined by the size of the focus [38]. It thus delivers spatially resolved information complementary to that obtained from the BioSAXS experiment, where the measured signal stems from an average of a large ensemble of organelles. Specifically in the case of the D2 sample, where the suspension SAXS data for surface and bulk properties in the low *q*-regime cannot be distinguished, selective probing with a nanofocus makes it possible to separate the two contributions.

### 2.5. Scanning electron microscopy of freeze-dried organelles

The freeze-dried and carbon-coated samples were imaged with a scanning electron microscope (Leo 1530 Gemini, Zeiss, Germany), using two different detector schemes: an in-lens configuration and an out-lens configuration. The in-lens detector, which is situated immediately above the sample in line with the incident electron beam, is capable of delivering images of superior resolution due its highly efficient collection of secondary electrons. The laterally displaced out-lens detector provides more topographic information, due to its pseudo-side illumination [39]. In both cases the acceleration voltage of the field-emission electron source was set to 3 keV.

## Results

### 3.1. Small angle X-ray scattering at ambient temperatures

**Figure 1:**
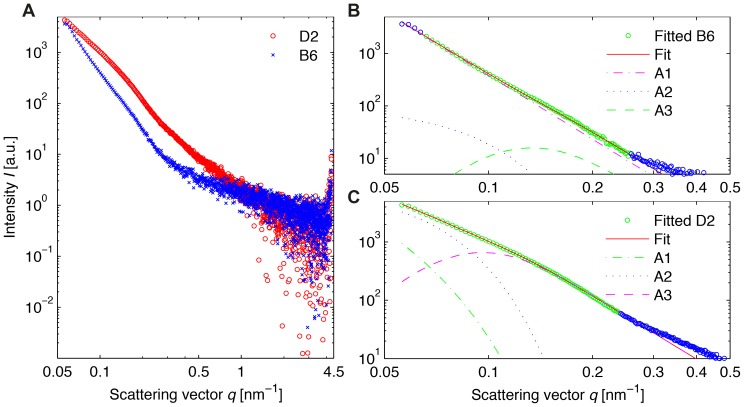
SAXS data for suspended melanosomes from C57BL/6J (B6) and DBA/2J (D2) mice. Melanosomes were exposed to photons at *E* = 12.8 keV for 10 s. (A) Comparison of scattering intensities for both phenotypes, in the range of 0.05 to 4.5 nm^−1^. (B) Analysis of B6 melanosome structure by fitting a model of independent scatterers to the *q*-interval 0.07 nm^−1^<*q*<0.27 nm^−1^. The resulting fitting parameters in the power law are *p*_1_ = 4.034±0.001 (small *q*-values), *p*_2_ = 3.887±0.118 (high *q*-values) und *R*_B6_ = 22.57±5.57 nm (radius of gyration of melanosomal subunits). (C) Analysis of D2 melanosome structure by fitting a model of dependent scatterers to data within the *q*-interval 0.05 nm^−1^<*q*<0.27 nm^−1^. The determined parameters are *p*_1_ = 2.56 (modified power law at small *q*-values), *p*_2_ = 3.60 (power law at high *q*-values) and *R*_D2_ = 31.32±1.71 nm (radius of gyration of melanosomal subunits). In (B) and (C) the terms that contribute to the fit model used are labeled A1, A2 and A3. The analyzed data points are shown in green.

We began our characterization of melanosome features with SAXS by analyzing purified pools of murine iris melanosomes at ambient temperatures (Fig. 1A). A comparative analysis was performed using samples from two widely utilized strains of mice C57BL/6J (B6) and DBA/2J (D2). The intensity plot in Fig. 1A illustrates that the scattering intensity for the D2 melanosomes is, in general, higher than that for their B6 counterparts. While the offsets in the SAXS curves could stem from slight differences in melanosome concentration in the illuminated capillary, the differences in the curve shapes across the sampled frequency range clearly reveal structural variances between the D2 and B6 organelles. In order to quantitatively characterize the features of the melanosome aggregates, we applied a simple model of independent scatterers to B6 melanosomes (Fig. 1B), and a more complex function based on dependent scatterers for D2 melanosomes (Fig. 1C).

For the B6 data, a fitted curve *I*_B6_(*q*) and the contributions of the three terms in Eq. (1) were calculated. The decline of the curve of the third term from Eq. (1) towards small *q*-values shows the effect of the rescaling with the error function erf(·). The results from the fitting process are *p*_1_ = 4.034±0.001, *p*_2_ = 3.887±0.118 and *R*_B6_ = 22.57±5.57 nm. The parameters resulting from fitting of the D2 data according to Eq. (3) are *p*_1_ = 2.56±0.02, *p*_2_ = 3.60±0.03 and *R*_D2_ = 31.32±1.71 nm.

According to Porod's law, *p*-values close to 4 can generally be attributed to homogeneous entities with smooth surfaces, and lower values to particles with a rather granular structure [40]. The values obtained above are the first indication that D2 melanosomes may have a more granular organization than their wild-type, B6 counterparts.

### 3.2. X-ray scattering at cryogenic temperatures

In order to obtain information complementary to that from the SAXS experiment described above, where many suspended organelles were illuminated simultaneously, and thus the specific structure of the D2 could not be disentangled with respect to surface and bulk information at low *q*-values, we performed a scanning experiment with single vitrified melanosomes. This made it possible to probe structural features with a nanofocus locally, and hence shed light on surface versus bulk features separately.

**Figure 2:**
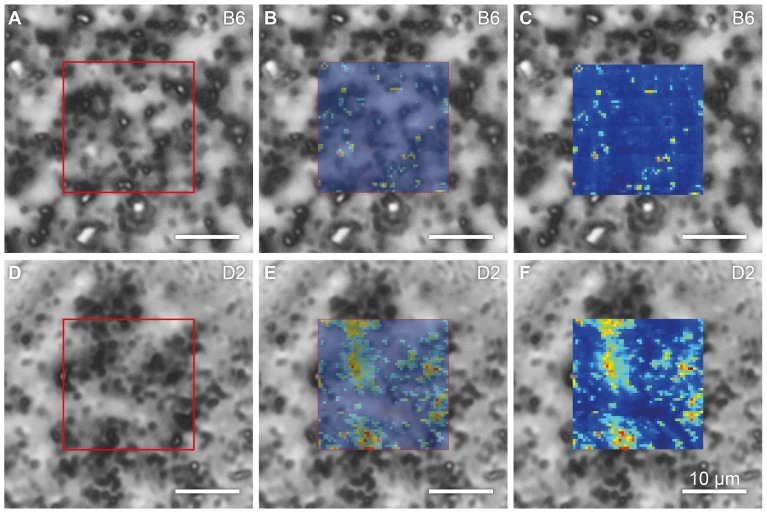
Correlation of maps generated by optical phase-contrast microscopy, cryo microscopy and darkfield cryo-STXM. Melanosomes of the genotypes C57BL/6J (B6) and DBA/2J (D2) are shown in tiles (A–C) and (D–F), respectively. In both cases, the sample is embedded in an amorphous ice matrix. The optical micrographs, recorded at 100 K, are superimposed with a semi-transparent (B and E) and an opaque (C and F) STXM map. Note that despite the fact that melanosome density is comparable in the two samples, the STXM images feature significant differences in signal intensity as well as the spatial distribution of that signal, revealing structural differences between the melanosomes of the two genotypes. The area of the scanned regions is 20.4×20.4 µm^2^.

Fields of vitrified B6 and D2 melanosome samples were imaged by optical microscopy and STXM, under cryogenic conditions (Fig. 2). An area of each sample was scanned with the X-ray beam, and a darkfield STXM image was produced by integrating the scattered photons in each raster point, plotting the intensities pixel-wise according to the beam position on the samples, and correlating optical and X-ray data to identify single melanosomes.

**Figure 3:**
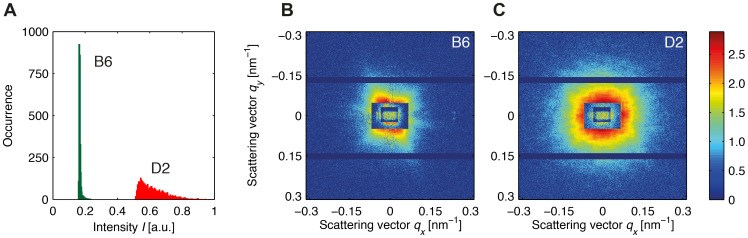
X-ray scattering data for cryogenically prepared melanosomes from C57BL/6J (B6) and DBA/2J (D2) mice. (A) Normalized intensity histograms derived from darkfield STXM maps of B6 and D2 samples like those shown in Fig. 2. The intensity of scatter for the D2 melanosomes is higher and has a broader distribution, than that for the B6 sample. The photon energy was 7.9 keV. (B) Sum of 13 background-corrected scattering events, i.e. *hits*, for a B6 sample. The scattering pattern is anisotropic. (C) Sum of 13 background-corrected scattering events for a D2 sample. The scattering pattern is isotropic. In (B) and (C), intensity is color-coded on a logarithmic scale. The horizontal bars in (B) and (C), which are devoid of signal, correspond to insensitive regions of the PILATUS, and separate the three detector modules. The artificial look of the regions in the centers is due to the stacking of two semi-transparent beamstops.

This analysis revealed qualitative differences between B6 and D2 melanosomes. Even though optical micrographs suggested that the two were of similar melanosome density (Fig. 2), the STXM maps of B6 melanosomes revealed a sparse scattering intensity compared to those of the D2 organelles. This effect is illustrated quantitatively in the plot presented in Fig. 3A. The graph consists of two histograms that represent the intensity distributions within the darkfield STXM maps shown in Fig. 2, with the intensities normalized to the maximum value in the D2 scan. Notably, the intensity of the signal in the B6 darkfield STXM map is lower than that in the D2 map. Moreover, the distributions of intensity in the two samples are of significantly different width. Whereas the distribution is rather broad in the case of the D2 organelles, it is almost binary in the case of the B6 melanosomes, with scatter at a particular signal intensity either detectable or not.

Analysis of 13 accumulated, background-corrected scattering events (Fig. 3B and 3C) of individual melanosomes from each strain indicated that B6 melanosomes exhibit anisotropic scattering patterns whereas D2 melanosomes have comparatively isotropic scattering. Because hard X-rays are far more influenced by three-dimensional structures and granularities on the nanoscale than the visible wavelengths used in optical microscopy, the observation of different scattering characteristics (*binary* vs. broad and directed vs. isotropic) again indicates that the B6 and D2 melanosomes have different levels of overall complexity.

### 3.3. Scanning electron microscopy of freeze-dried organelles

**Figure 4:**
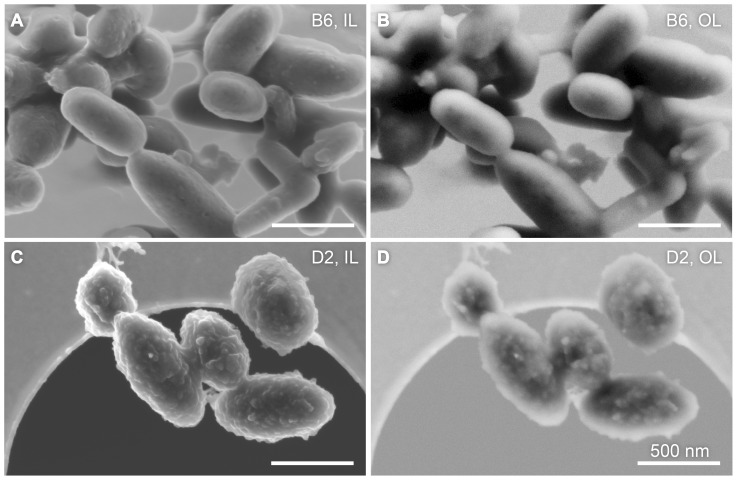
SEM images of freeze-dried C57BL/6J (B6) and DBA/2J (D2) melanosomes. The left column contains images recorded in the in-lens detector configuration (IL) and the right column shows micrographs acquired in the out-lens configuration (OL). A direct comparison shows that B6 melanosomes (A and B) have a rather smooth and featureless surface, whereas the D2 organelles (C and D) have an irregular surface. The samples were subjected to the identical preparation protocol. All scale bars represent 500 nm.

Fig. 4 shows a vis-à-vis comparison of SEM images of freeze-dried B6 and D2 melanosomes. Due to the small depth of penetration by 3-keV electrons, the micrographs are exclusively surface sensitive. The magnification factors were identical. Images recorded with the in-lens and out-lens detector are marked with “IL” and “OL”, respectively. As expected, the in-lens scheme yielded images of superior resolution. Regardless of the detection scheme, however, the micrographs reveal significant differences in surface morphology between both melanosome phenotypes. This experiment suggests that the D2 melanosomes differ from their B6 counterparts in that they have a more complex surface structure.

## Discussion

The three modes of analysis used here – SAXS analysis of many thousand suspended melanosomes, SAXS analysis of single vitrified melanosomes and SEM from freeze-dried samples – provide complementary information that can be used to derive a structural model of melanosomes from both strains of mice. According to the calculated fitting parameters from the suspension SAXS data, the surface of the B6 melanosomes is smooth (*p*_1_ = 4.0), with scales corresponding to *q*<0.1 nm^−1^. These organelles are made up of densely packed subunits, which themselves have a rather smooth surface (*p*_2_ = 3.9) and are roughly 45 nm in diameter (*R*_B6_ = 22 nm). These values lie within the slope interval that is specific for so-called surface fractals (3<*p*<4). In contrast, the D2 melanosomes appear to exhibit a looser, granular ultrastructure, as indicated by a smaller *p*-value (*p*_1_ = 2.6), i.e. one within the slope regime for volume fractals (1<*p*<3) [32], [34], [41]. Their subunits also seem to be coarser than those of the B6 melanosomes, with a radius of gyration of approximately 60 nm (*R*_D2_ = 31 nm); the surfaces of these small building blocks appear to be rougher than those of the B6 melanosome subunits (*p*_2_ = 3.6).

Our X-ray scattering results on individual melanosomes from the cryogenic experiment add another layer of confidence to the above-described conclusions drawn from the ensemble SAXS data gathered at 10°C. Moreover, they provide additional information with regard to the surface structures of loose aggregates like the D2 melanosomes. Such information cannot be gleaned from the BioSAXS experiment, as scattering from internal granular structures cannot be distinguished from that occurring at the surface.

In interpreting results from the scattering of single vitrified organelles, it is important to keep in mind that the focal spot used in this experiment (measures 400×200 nm^2^) is on average smaller than a single melanosome (compare to Fig. 4). When the central part of an object without any, or with only little, internal structure is probed by a penetrating X-ray beam that is smaller than the object, only few photons are deflected from the optical axis. The vast majority passes the sample undeflected, and hence the darkfield intensity is low. However, when the beam impinges on the edge of an object with a smooth surface, the photons are scattered in the perpendicular direction. In such a case, the darkfield signal is high and the two-dimensional scattering patterns shows streaks rather than isotropic intensity distributions. In contrast, objects that have a rough surface and granular internal structures will show high scattering intensities with an isotropic distribution.

These two outcomes are illustrated in Figs. 3B and C, indicating that the melanosomes contain a disordered aggregate of scatterers with a well defined (B6) or more diffuse (D2) edge. The B6 organelles are characterized by low darkfield intensities and directed scattering, suggesting that they have a smooth surface and homogeneously distributed internal material. The D2 samples, on the other hand, show high darkfield intensities and isotropic scattering. Thus, these melanosomes differ significantly from their B6 counterparts, featuring both a rough surface and a rather granular internal structure. This conclusion about surface roughness is further supported by the results of SEM analysis (Fig. 4), as the B6 samples look rather smooth but D2 melanosomes exhibit a coarse and rough appearance.

This investigation is subject to several known caveats and limitations that must be taken into account when interpreting the data. Firstly, by definition, the radius of gyration does not precisely match the dimensions of a physical object, but rather the standard deviation of the mass distribution. Therefore, our use of the radius of gyration as a reasonable estimate of the physical size of the ultrastructural subunits assumes that most melanosomal subunits are spherical in shape. Although observations of spherical melanin aggregates that are visible during early stages of melanosome maturation [42] have led to a belief that mature melanosomes have spherical organization, this remains an assumption. Secondly, despite the fact that cryogenic and suspended vitrified samples are prepared with only minimum fixation, artifacts cannot be entirely excluded. For example, it is possible that the structural integrity of D2 melanosomes is low and that they do not necessarily exhibit the characteristics described here while in the living organism, as structural weakness could make them sensitive to mechanical stress during their extraction and centrifugation.

While the nature of the 40–60 nm subunits within melanosomes remains speculative, their existence has been corroborated by multiple studies. Studies using atomic-force microscopy have detected substructures of similar size at the surface of melanosomes from human hair [15], the human iris [43] and the human retinal pigment epithelium [44], as well as in neuromelanin [45]. Also, TEM has made it possible to observe structural subunits in the context of reduced melanin content. Examples of such scenarios include: early developmental stages of melanin biogenesis in normally pigmented cells [46]; conditions in which melanosomes are degraded *in vivo* by macrophages [47]; and melanosomes treated *ex vivo* with ammonia [48]. Because electron-dense melanin is known to fill melanosomes, it is logical to assume that the subunits consist predominantly of the melanin polymer. By extension, if the surface melanin imaged by atomic-force microscopy is constrained into 30–60 nm subunits, the prediction is that all melanin within melanosomes exists in this configuration.

These findings regarding the melanosomal ultrastructure are of potential biological relevance. The two inbred strains of mice used in this study, B6 and D2, have known differences in pigment-cell biology, including different coat colors [49] and different susceptibilities to pigment-related eye disease [50]. Among the many genetic differences between these strains that could potentially impact pigmentation are mutations in four genes of known relevance: *Myo5a* (commonly known as dilute) [51], *Tyrp1* (commonly known as brown) [52], [53], *a* (commonly known as nonagouti) [54], and *Gpnmb* [55]. These genes are thus candidates for the underlying strain-dependent melanosomal features we have identified. Moreover, three of these are known to influence pigmentation [56], as reflected by the naming of the so-called DBA-derived strains (dilute, brown, nonagouti), and the remaining gene (*Gpnmb*) encodes a melanosomal protein of particular relevance to melanosomes of the iris [12], [50], [55].

Inbred mice of the D2 strain spontaneously develop pigment dispersion syndrome due to a synergistic effect in mutations of the *Gpnmb* and *Tyrp1* genes [55]. It has been hypothesized that D2 melanosomes evoke a toxic process linked to melanin synthesis [57], [58] that causes damage to vital cells [10]. The toxicity could well involve altered melanosomal morphology – consistent with our findings regarding D2 melanosomes. Further experiments are required to test whether the strain-dependent melanosomal features revealed by our SAXS analysis are caused by these mutations, or by other genetic variants that remain to be identified.

## Conclusions

The SAXS analysis presented here revealed structural differences within melanosomes from mice of different genetic backgrounds. This is the first demonstration of the usefulness of small angle X-ray scattering in organelle research, and we expect that many additional applications of this type will follow. The great advantage of this indirect approach over direct imaging techniques is its averaging of data from a large ensemble of entities that simultaneously contribute to the measured signal. This puts the measurements on a strong statistical foundation. Notably, the use of suspended organelles does not require sample preparation steps beyond their extraction from mice, thus effectively circumventing potential artifacts of preparation.

The biological result from the scattering data obtained from vitrified samples and SEM images verified the hypothesis that melanosomes extracted from the D2 mice differ with respect to their internal and external structures from their wild type B6 counterparts. Surface and volume effects were disentangled, such that coarsening of the internal structure in the D2 melanosomes became obvious.
