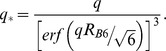# Correction: Nano-Scale Morphology of Melanosomes Revealed by Small-Angle X-Ray Scattering

**DOI:** 10.1371/journal.pone.0101746

**Published:** 2014-07-01

**Authors:** The PLOS ONE Staff

In the Methods section, there is an error in the second equation under the sub-heading “Small angle X-ray scattering at ambient temperatures.” Please view the complete, correct equation here: